# “Rhizoponics”: a novel hydroponic rhizotron for root system analyses on mature *Arabidopsis thaliana* plants

**DOI:** 10.1186/s13007-015-0046-x

**Published:** 2015-01-23

**Authors:** Laura Mathieu, Guillaume Lobet, Pierre Tocquin, Claire Périlleux

**Affiliations:** Department of Life Sciences, Laboratory of Plant Physiology, PhytoSYSTEMS, University of Liège, Liège, Belgium

**Keywords:** Roots, Phenotyping, Rhizotron, Hydroponic

## Abstract

**Background:**

Well-developed and functional roots are critical to support plant life and reach high crop yields. Their study however, is hampered by their underground growth and characterizing complex root system architecture (RSA) therefore remains a challenge. In the last few years, several phenotyping methods, including rhizotrons and x-ray computed tomography, have been developed for relatively thick roots. But in the model plant *Arabidopsis thaliana*, *in vitro* culture remains the easiest and preferred method to study root development, which technically limits the analyses to young seedlings.

**Results:**

We present here an innovative design of hydroponic rhizotrons (rhizoponics) adapted to *Arabidopsis thaliana*. The setup allows to simultaneously characterize the RSA and shoot development from seedling to adult stages, i.e. from seed to seed. This system offers the advantages of hydroponics such as control of root environment and easy access to the roots for measurements or sampling. Being completely movable and low cost, it can be used in controlled cabinets. We chose the case of cadmium treatment to illustrate potential applications, from cell to organ levels.

**Conclusions:**

Rhizoponics makes possible, on the same plants of Arabidopsis*,* RSA measurements, root sampling and characterization of aerial development up to adult size. It therefore provides a valuable tool for addressing fundamental questions in whole plant physiology.

**Electronic supplementary material:**

The online version of this article (doi:10.1186/s13007-015-0046-x) contains supplementary material, which is available to authorized users.

## Background

Plant roots are responsible for nutrient and water uptake and are thus critical components of the overall plant productivity [1]. The root system architecture (RSA) is determined by both endogenous factors (reviewed in [2]) and environmental constraints such as nutrient availability [3,4]. Therefore, the understanding of the mechanisms regulating RSA is important for future crop improvement [5,6].

Arabidopsis (*Arabidopsis thaliana*) remains the most widely used plant model for studying fundamental processes in plant biology, while there is an increasing interest for transposing and acquiring further knowledge on economically relevant plants such as maize (*Zea Mays)* or rice (*Oryza sativa*) (Additional file 1). Regarding RSA studies, the relative simplicity of Arabidopsis root system makes it an ideal case to study the impact of endogenous traits and/or exogenous factors on root development, such as the genetic variation in plant response to nitrate supply [7] or the toxicity of cadmium on root and shoot development [8].

As a largely mastered plant lab technique, *in vitro* culture is the most common method used to study RSA in Arabidopsis. Seedlings are grown in vertically placed Petri plates and hence the roots develop along the surface of the medium, in a 2-D space suitable for image-based analyses, and can be easily harvested. However, several disadvantages are intrinsically linked to *in vitro* culture. Firstly, roots are exposed to light, which has been shown to influence their development [9]. Secondly, the confined atmosphere limits gas exchanges and metabolism, so that exogenous sucrose is frequently supplied in the medium. Finally, the size of the Petri plates limits the studies to young plants (2 to 3 weeks).

In the last few years, alternative methods have emerged to phenotype RSA in the laboratory (Table 1). Germination on paper (‘pouches’) [10,11] has become popular for the study of RSA of crop plants. However it is not suitable for fine root system analyses (such as the one of Arabidopsis*)* because of the difficulty to distinguish the roots from the fibrous background. Soil-based 2D- or 3D-methods, such as rhizotrons [12,13] or x-ray tomography [14], have been adapted to Arabidopsis. A transparent solid medium was developed to facilitate the observations [15]. Major drawbacks of the existing soil-like methods are their reduced flexibility and the fact that the roots are not accessible for sampling.

**Table 1:** **Feature comparison of existing culture setups**

**Methods**	**Root tissues collection**	**RSA analysis**	**Long time-course analysis**	**Nutrients control**	**Suitable for Arabidopsis**	**Heterogenous root environment**
Rhizotron	NO	YES	YES	YES	NO	YES
Pouch	YES	YES	NO	YES	NO	YES
Transparent soil	NO	YES	NO	YES	YES	YES
Hydroponics	YES	NO	YES	YES	YES	NO
Rhizoponics	YES	YES	YES	YES	YES	NO

Hydroponics is frequently used for studies requiring control of nutrients and accessibility to the root system. Hydroponics is appropriate for cultivation of the plants throughout their entire life cycle, does not limit gas exchanges, and allows to perform independent experiments in reproducible root-environment conditions. However quantification of root system architecture is not possible in such systems due to root tangling in the liquid medium. This is an even more acute issue with small plants, such as Arabidopsis, having a fine root system [16,17].

An efficient hydroponic device was previously developed in our lab for synchronous growth and flowering of Arabidopsis [16]. Here we present an innovative system (named “rhizoponics”) that combines the advantages of hydroponics and rhizotrons to study RSA of Arabidopsis adult plants. Roots are grown in 2D, without any physical constraint, on a framed nylon support immersed into the nutrient solution. Rhizoponics is readily amenable to image analysis and allows sampling of selected roots in controlled conditions.

## Results and discussion

### Setup description

**Figure 1:**
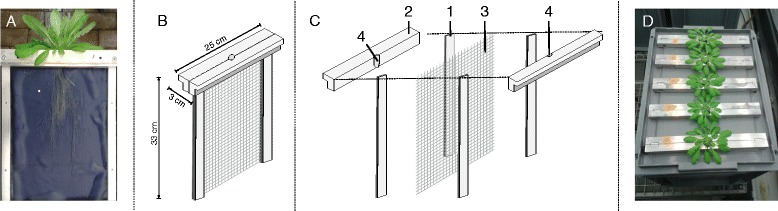
**Schematic representation of the rhizoponics setup. A**. Picture of the rhizoponics setup. A blue paper was placed behind the setup to increase the contrast. **B**. Assembled setup. **C**. Exploded view of the setup. 1. Lateral vertical element. 2. Top horizontal element. 3. Nylon mesh. 4. Hole for the Araponics seed holder. **D**. Full system picture.

The rhizoponics setup is made of a nylon fabric (mesh of 0.5 mm^2^) that is maintained in an aluminium frame of 335 × 250 mm (Height x Width) (Figure 1). The frame hangs in a tank filled with nutritive solution and is therefore slid into a slot of the tank cover. The tank and its cover are dark to prevent light exposure of the roots and the nutritive solution. The top side of the U-frame is larger (30 mm) that the cover slot in order to block the frame. It bears a hole in its middle, designed to insert an Araponics seed holder (http://www.araponics.com/) and supports the aerial rosette. Rhizoponics blueprints and 3D files are provided in the Additional files 2 and 3. Piece profiles could be easily adapted to other homemade seed starting systems, e.g. pierced microcentrifuge tubes [16,17], or to other hydroponics tanks.

Rhizoponics were designed to be light in weight and easily assembled. Aluminium was chosen as a building material for (i) its rigidity, (ii) its anti-corrosive properties and (iii) the possibility to sterilize the frames. However, we believe that any rigid plastic material could be used, opening the door to the use of 3D printing technologies.

### Selection of the fabric

Various fabrics were tested. The size of the mesh appeared to be the critical point: a too narrow mesh allows the thin roots of Arabidopsis to penetrate into the fabric and thus makes the image analysis impossible. Conversely, a wide mesh does not provide an efficient support to untangle the roots. The selected fabric (black polyamide tulle netting, or Crinoline, 112 g m^-2^, http://www.whaleys-bradford.ltd.uk/nylon-crinalin-black) has a mesh of 0.5 mm^2^ and is perfectly adapted to Arabidopsis RSA. The fabric can be cleaned, autoclaved and recycled between experiments.

### Pre-culture

The seed holders are filled with agar as described in [16] and the seeds can be sown directly on top of it, in the rhizoponics setup. However, we observed that a pre-germination step in a standard Araponics tank allowed more synchronous germination and subsequent selection of homogeneous seedlings before transfer into the rhizoponics.

### Image acquisition

**Figure 2:**
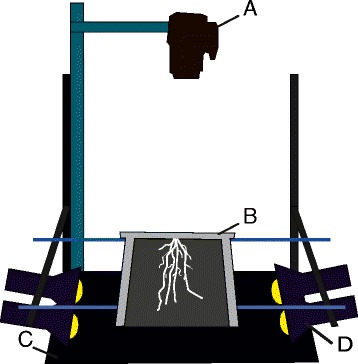
**Schematic representation of the imaging setup adapted to rhizoponics setup. A**. Camera. **B**. Rhizoponics. **C**. Dark background. **D**. Indirect light.

Different setups were tested to acquire pictures of the root system. The main challenge was to find the right light source/background combination in order to distinguish the roots from the mesh. Direct lightning (light source on the same side as the camera) was not satisfactory as it did not discriminate the roots from the mesh. Moving the light source to the sides of the rhizoponics (raking lightning) greatly improved the contrast by highlighting the texture differences due to the presence of roots. However, this method allows the observation of the roots at one side of the mesh only. Direct backlighting was tested using an illuminated box, but the roots lacked contrast against the bright background. Finally, the best setup was based on indirect backlighting. The roots were placed above a black surface towards which the light source was directed (Figure 2). Raw pictures are provided in the Additional file 4.

### Rhizoponics setup enables a precise quantification of root and shoot development

**Figure 3:**
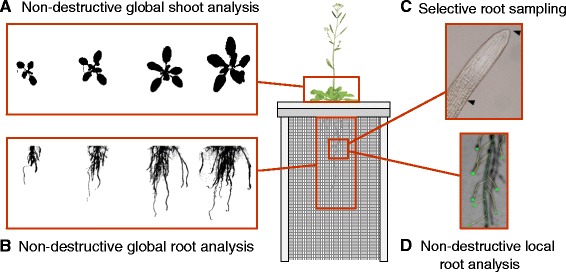
**Illustration of global (A, B) and detailed (C, D) analyses that can be performed with the rhizoponics setup.**

The rhizoponics setup enables the simultaneous and non destructive observation of root and shoot development during the whole plant cycle (Table 2). On the shoot side, global growth estimators such as the projected rosette area or diameter can be easily followed (Figure 3A). On the root side, similar global estimators can be obtained (Figure 3B) as well as local features (lateral root density, length of unbranched root apical zone, lateral root length, (Figure 3D). In addition to these image-based analyses, the setup enables easy and local root sampling, without damaging the rest of the plant. Time course experiments are thus feasible, e.g. for microscopic observations or gene-expression analyses (Figure 3C).

**Table 2:** **Non-exhaustive list of measurements enabled by the rhizoponics setup**

**Organ**	**Measurements**	**Units**	**Destructive**
Shoot	Projected rosette area	cm^2^	No
	Rosette diameter	cm	No
	Rosette convex hull area	cm^2^	No
	Leaf count	-	No
	Rosette fresh/dry weight	g	Yes
Root	Projected root surface	cm^2^	No
	Root system depth	cm	No
	Root system width	cm	No
	Root system convex hull area	cm^2^	No
	Lateral root density	root/cm	No
	Length of unbranched apical root zone	cm	No
	Lateral root length	cm	No
	Microscopy analyses	-	Partial
	Reporter gene observation	-	Partial
	Root system fresh/dry weight	g	Yes

### Case study: effect of cadmium toxicity

As a proof of concept, we analysed the effect of cadmium (Cd) on Arabidopsis development. Cd is a heavy metal which is known to be toxic and limits shoot and root growth. However it is not lethal for the plant and the reproductive phase occurs normally [8,18].

**Figure 4:**
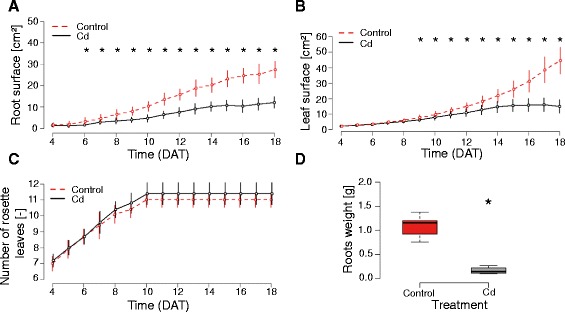
**Effect of cadmium on global root and shoot measurements. A**. Projected root surface. **B**. Projected rosette surface. **C**. Number of visible rosette leaves. **D**. Root weight measured 18 days after start of treatment. Stars indicate a significant difference between treatments (t-test, p-value < 0.05, n = 10).

Taking advantage of the rhizoponics setup, root and shoot growth kinetics were followed simultaneously during the whole life cycle of plants treated with 10 μM Cd. We observed a highly significant inhibition of root and shoot growth following Cd-treatment (Figure 4A & B). The effect of the treatment was first observed on the root system (6 days after start of treatment, DAT) and later on the shoot (9 DAT). We also observed, in line with previous results [18], that although the size of the rosette was strongly reduced in Cd-treated plants, the leaf apparition rate was not significantly affected (Figure 4C) indicating that individual leaf expansion - and not leaf number - was limited.

**Figure 5:**
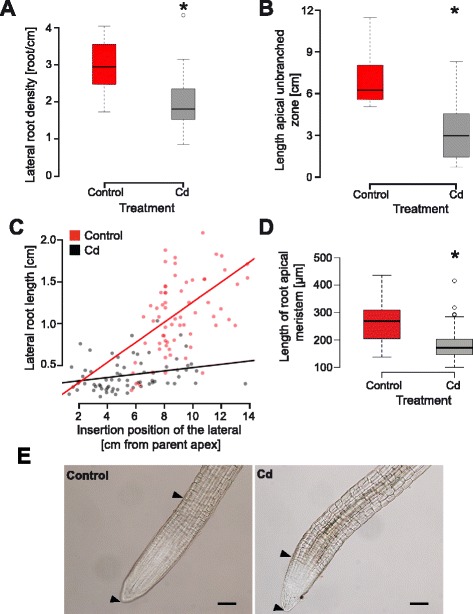
**Effect of cadmium on lateral root growth measured 18 days after start of the treatment. A**. Lateral root density (n = 10). **B**. Length of the apical unbranched zone (n = 10). **C**. Lateral root growth, illustrated by the relationship between the insertion position (distance from the parent root apex) and the length of the lateral roots. **D**. Length of the lateral root apical meristem (nmock = 102, nCd = 100). Significant differences (t-test, p-value < 0.05) are indicated by stars. **E**. Pictures of root apical meristem of control (left) and cadmium treated plants (right). Black lines represent 100 μm. Arrowheads indicate the limits of the root apical meristem.

One of the major advantages of our rhizoponics setup is to enable the analysis of both global traits (such as those presented in Figure 3) and local root system parameters. In this example, we analysed the effect of Cd on the lateral root density, the length of the unbranched apical zone (LAUZ) and the lateral root growth. Our measurements revealed a significant negative effect of Cd on all parameters (Figure 5), indicating a strong inhibition of lateral root development.

We harvested lateral root tips to determine the size of their meristem and observed that they have a smaller length in Cd-treated plants than in controls (Figure 5D & E). This difference possibly explains the overall reduced growth of the lateral roots.

At the end of the experiment, Cd-treated plants had developed inflorescences and produced siliques. Root systems were harvested for final biomass quantification. The fresh root weight of treated plants was reduced by four in comparison with control plants (Figure 4D), which is consistent with the literature [8]. Thanks to RSA measurements, we determined that root biomass was best correlated with the total surface of the root system (Pearson correlation = 0.94). This result further shows the efficiency of rhizoponics to untangle the root system and provide relevant pictures of it.

## Conclusion

We presented here a novel “rhizoponics” system that allows time-lapse studies of root system architecture and aerial part in Arabidopsis, up to mature stages. This system combines the advantages of hydroponics and rhizotrons. On one hand, roots grow in a controlled environment, ensuring high reproducibility of the results. On the other hand, a neutral support untangles the roots and so allows both global (e.g. total size, rooting depth) and local (e.g. lateral density, lateral length) analyses. Root sampling is easy, typically for molecular and microscopic analyses. As such, the rhizoponics setup opens new avenues for root/shoot researches.

## Material and methods

### Plant material and growing conditions

Arabidopsis (*Arabidopsis thaliana*, Col-0) seeds were stratified during 3 days on wet filter paper at 4°C in darkness. Thereafter, they were sown on Araponics (http://www.araponics.com/) seed-holders filled with 0.66% agar (Kalys HP697). Hydroponic solution was prepared with Flora Series fertilizers (FloraBloom, FloraMicro and FloraGro; GHE, France; 0.5 mL l^-1^ each). Seedlings were grown in 16-hour long days, at a (fluorescent) light fluence rate of 60 μmol m^-2^ s^-1^ (PAR), day/night temperature of 20°C and air relative humidity of 70%.

Two weeks after sowing, seedlings with 1-cm primary root were selected for transfer - together with their supporting seed-holder - into rhizoponics (1 plant/rhizoponic).

Rhizoponics were suspended in a plastic box (EURONORM 400X300X320 mm - STANDARD) filled with the same nutrient solution as described above. For Cd treatment, Cd- sulfate (3CdO4S.8H2O, Fluka 20920) was directly added into the box to obtain a final concentration of 10 μM.

### Measurements

Leaves were counted every day starting 4 days after seedling transfer into rhizoponics. Pictures of both the root system and the shoot were also taken daily. Fresh root biomass was weighed at the end of the experiment. Prior to weighting, the roots were blotted on a tissue to remove the excess of water. The length of the root apical meristem was measured from the root tip, up to the beginning of the elongation zone with the imaging software *NIS*-Elements 3.20 [19].

### Image collection

Shoot and root parts were photographed with a consumer CCD camera (Canon EOS 1100D, lens Canon EF 50 mm f/2.5). Aerial parts were imaged from above (top views), without removing the plants from the growing setup. For root imaging, rhizoponics were taken out of the tanks and placed horizontally, in a dark room, 15 cm above a black surface lightened with 4 incandescent bulbs of 40 W, placed in two rows (Figure 2). The camera was placed above the rhizoponic and aperture size and speed were set such as the acquired image was slightly under-exposed. ISO value was set to 200.

### Image analysis

Root and shoot images were analysed using custom build ImageJ plugins and macros [20].

For shoot images, color images were split, based on their Hue-Saturation-Value (HSB) channels. The rosette segmentation was performed on the Hue channel since it provided an optimal contrast between the plant (green) and the growing setup colour (both the frame and the box are grey) [21]. The diameter, projected surface and convex hull surface were extracted from the mask using native ImageJ functions.

**Figure 6:**
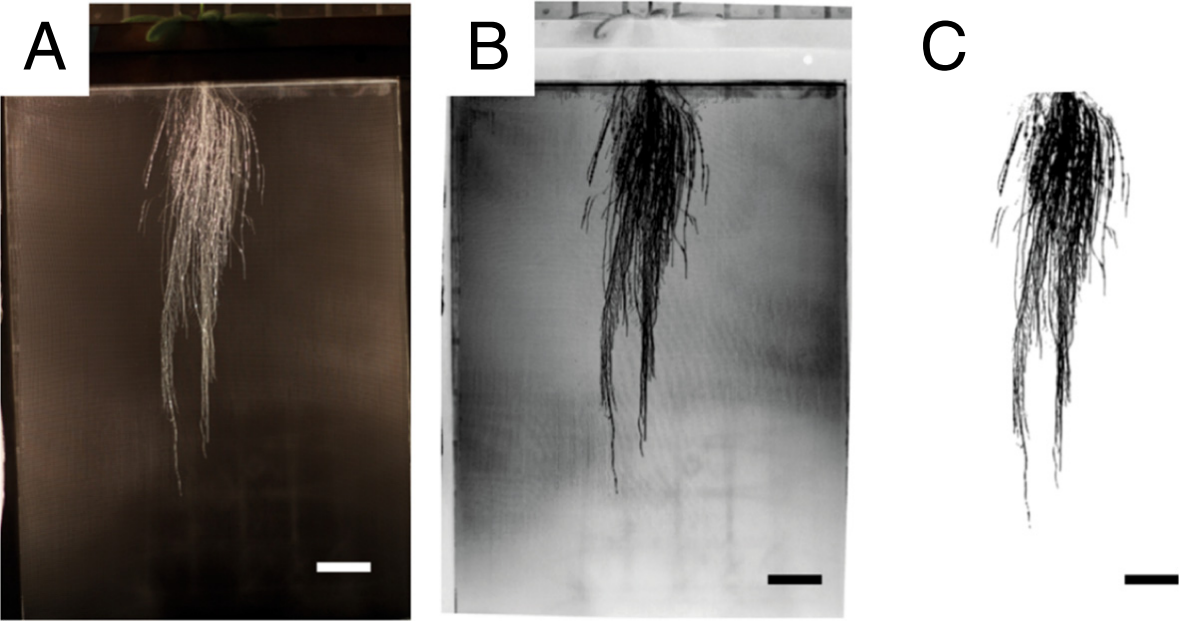
**Root image treatment. A**. Original color image. **B**. Grayscale image (Value channel from the HSV stack) obtained from **A**, with an increased contrast. **C**. Root system mask obtained from **B**, for quantitative analyses Bars = 3 cm.

Two different treatments were performed on the root images, depending on the type of analysis required (local or global). For the local analysis, color images (Figure 6A) were split based on their HSV value. In this case, unlike the shoot, the Hue channel did not discriminate the roots from the mesh. The Value channel was used instead. The Value channel images (Figure 6B) were used for the local analysis, while the segmented images (Figure 6C) were used for the global analysis. SmartRoot [22] was used to analyse selected roots in the images. From these roots, we computed the length of the unbranched apical zone (LAUZ) and the lateral root density. For the global analysis, the width, height, projected surface and convex hull surface were computed from the mask.

The image analysis scripts (ImageJ macros) are freely available on GitHub (https://github.com/guillaumelobet/rhizoponics).

## Additional files

Additional file 1:
**Number of publications on different plant species in 2013.** Vernacular names were used for search in abstracts, titles and keywords of papers referenced in the Biochemistry, Genetics and Molecular Biology section of the Scopus© database. Additional file 2:
**Rhizoponics blueprints.**
Additional file 3:
**Rhizoponics blueprints (readable with FreeCAD 3D:** **http://www.freecadweb.org/** **).**
Additional file 4:
**High definition picture of RSA 13 days after the transfer (DAT).** A and B,control plants. C and D,Cd-treated plants.
