# Supplementary material for: “Rhizoponics”: a novel hydroponic rhizotron for root system analyses on mature Arabidopsis thaliana plants
Source: Plant Methods. 2015 Jan 23;11:3. doi: 10.1186/s13007-015-0046-x (PMC4318444; doi:10.1186/s13007-015-0046-x)
Supplement: Additional file 1: — Number of publications on different plant species in 2013. Vernacular names were used for search in abstracts, titles and keywords of papers referenced in the Biochemistry, Genetics and Molecular Biology section of the Scopus© database. [file 13007_2015_46_MOESM1_ESM.docx]

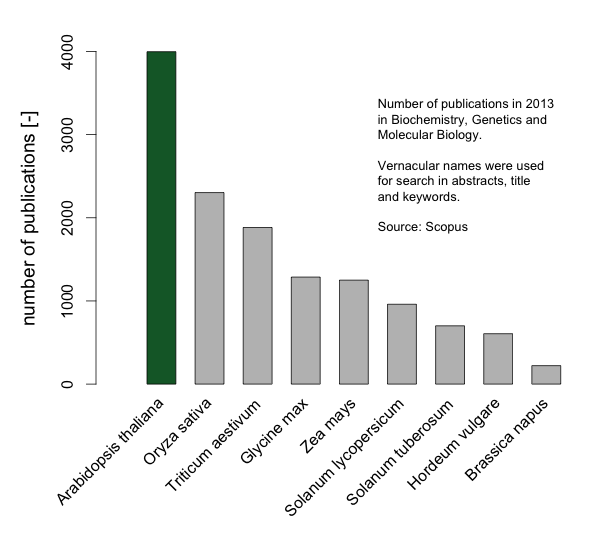


**Additional file 1: Number of publications on different plant species in 2013.** Vernacular names were used for search in abstracts, titles and keywords of papers referenced in the Biochemistry, Genetics and Molecular Biology section of the Scopus© database.
