# Supplementary material for: “Rhizoponics”: a novel hydroponic rhizotron for root system analyses on mature Arabidopsis thaliana plants
Source: Plant Methods. 2015 Jan 23;11:3. doi: 10.1186/s13007-015-0046-x (PMC4318444; doi:10.1186/s13007-015-0046-x)

Rhizoponic setup

Laura Mathieu, Guillaume Lobet, Pierre Tocquin and Claire Périlleux
PhytoSYSTEMS, Université de Liège, Belgium

All measures are in millimetres

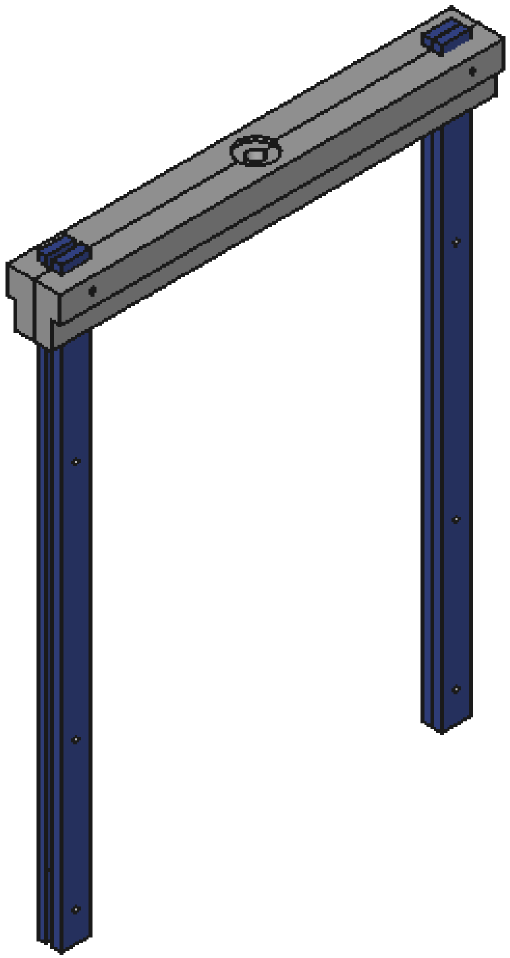

Upper piece x2

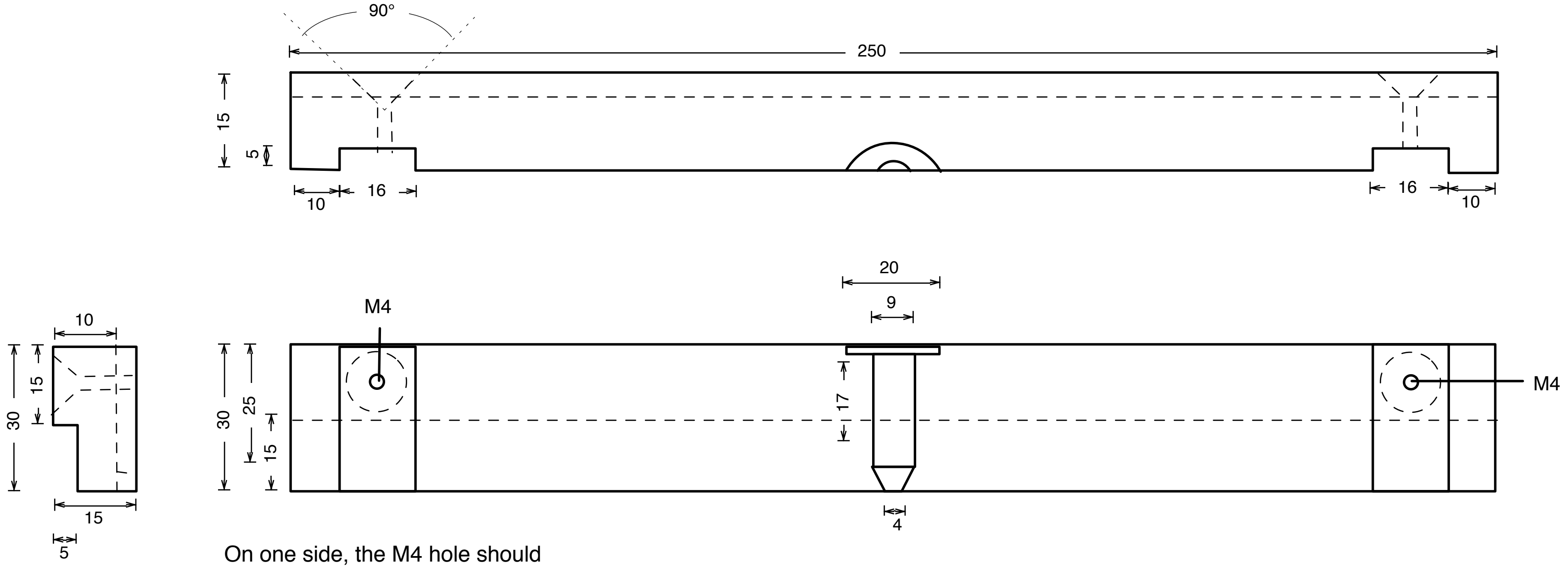

On one side, the M4 hole should have a screw thread.

Side piece

x4

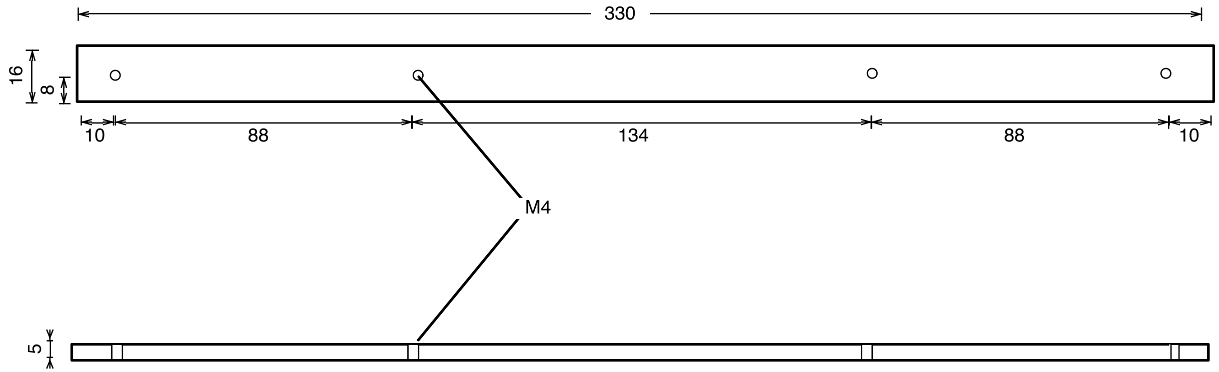

Supplement: Additional file 2: — Rhizoponics blueprints. [file 13007_2015_46_MOESM2_ESM.pdf]
