# Supplementary figures and images for: “Rhizoponics”: a novel hydroponic rhizotron for root system analyses on mature Arabidopsis thaliana plants
Source: Plant Methods. 2015 Jan 23;11:3. doi: 10.1186/s13007-015-0046-x (PMC4318444; doi:10.1186/s13007-015-0046-x)

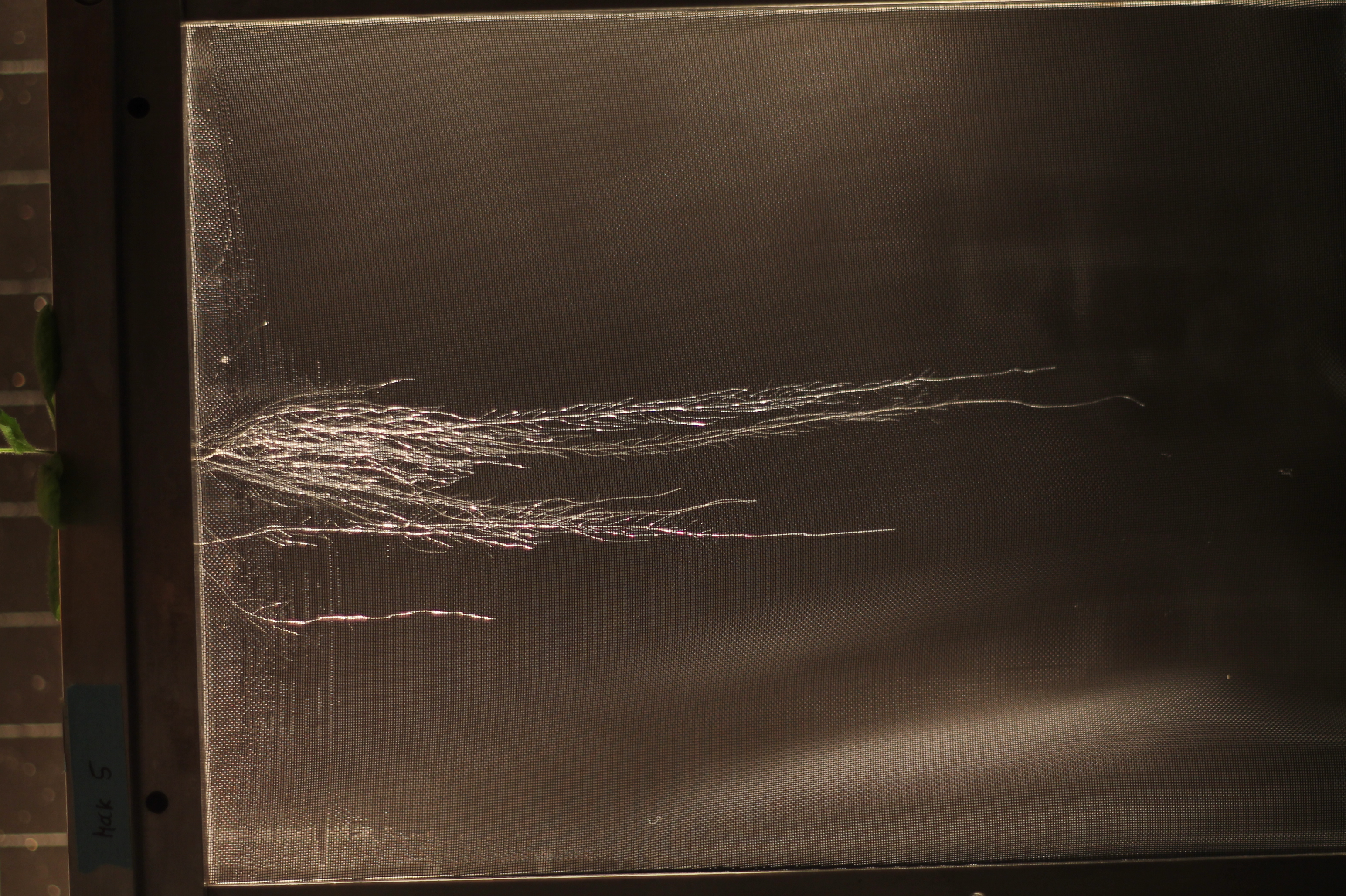

Supplement: Additional file 4: — High definition picture of RSA 13 days after the transfer (DAT). A and B,control plants. C and D,Cd-treated plants. [file 13007_2015_46_MOESM4_ESM.zip › Additional file 4 B.JPG]

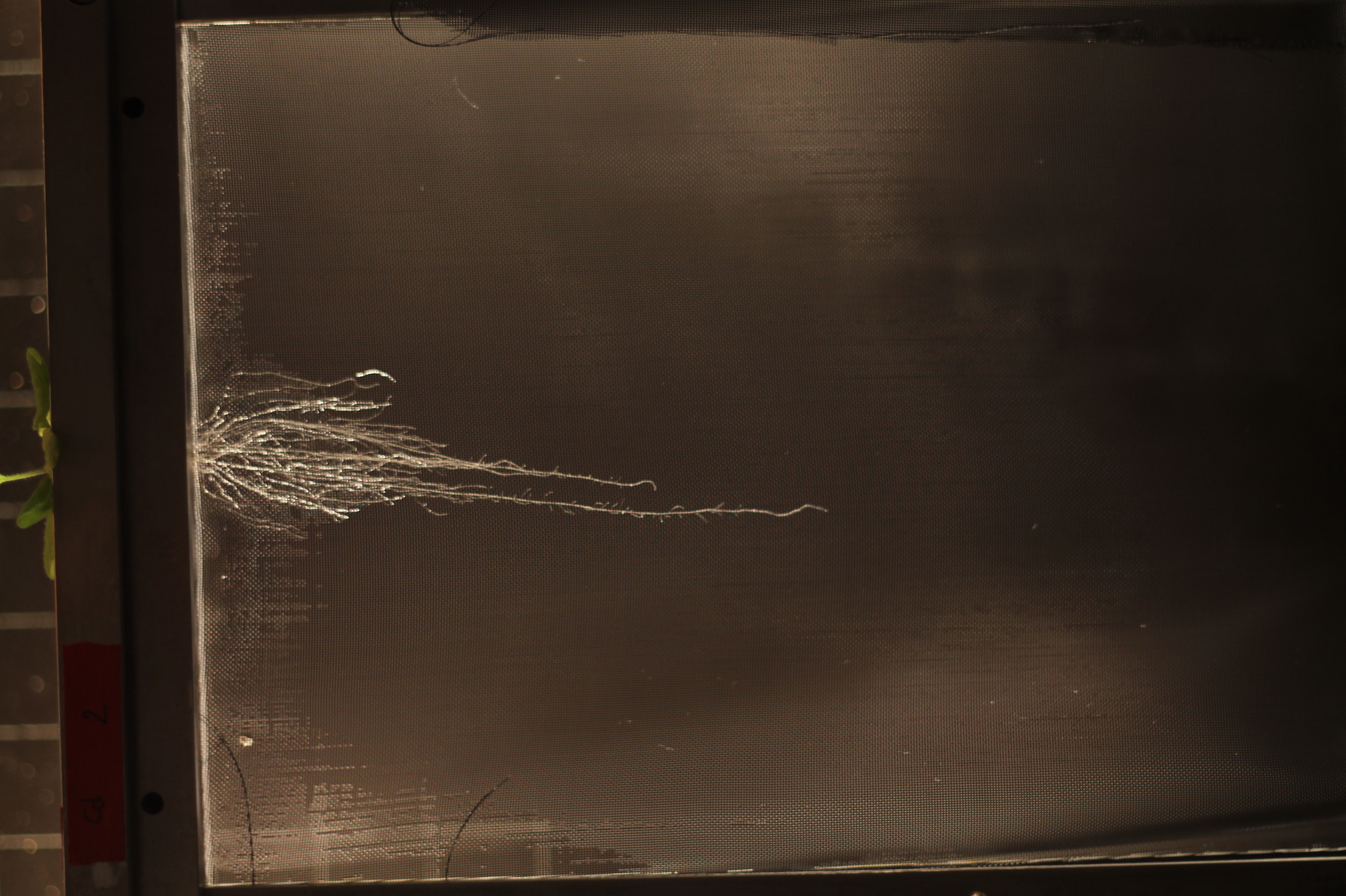

Supplement: Additional file 4: — High definition picture of RSA 13 days after the transfer (DAT). A and B,control plants. C and D,Cd-treated plants. [file 13007_2015_46_MOESM4_ESM.zip › Additional file 4 C.JPG]

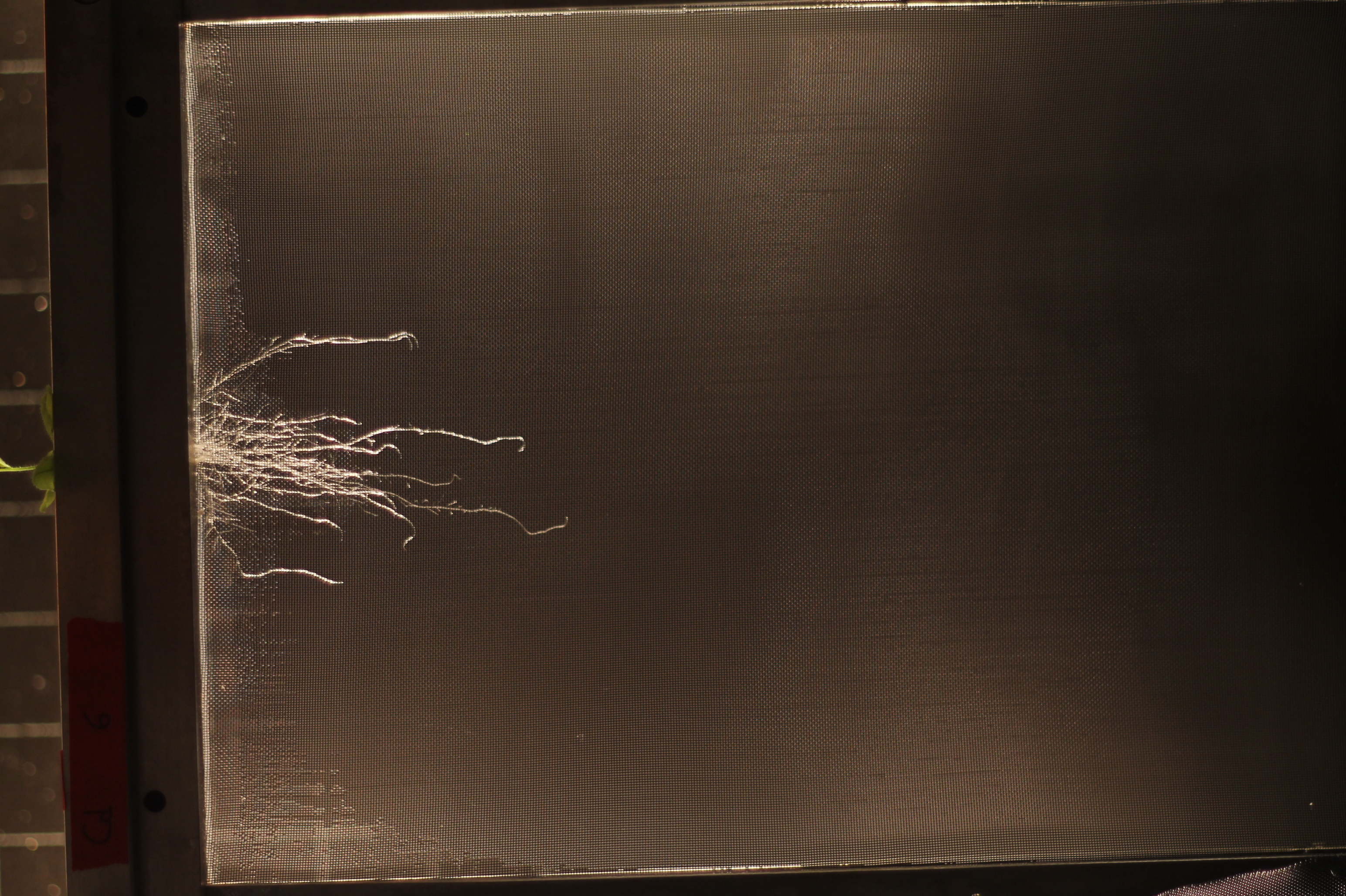

Supplement: Additional file 4: — High definition picture of RSA 13 days after the transfer (DAT). A and B,control plants. C and D,Cd-treated plants. [file 13007_2015_46_MOESM4_ESM.zip › Additionnal file 4 D.JPG]

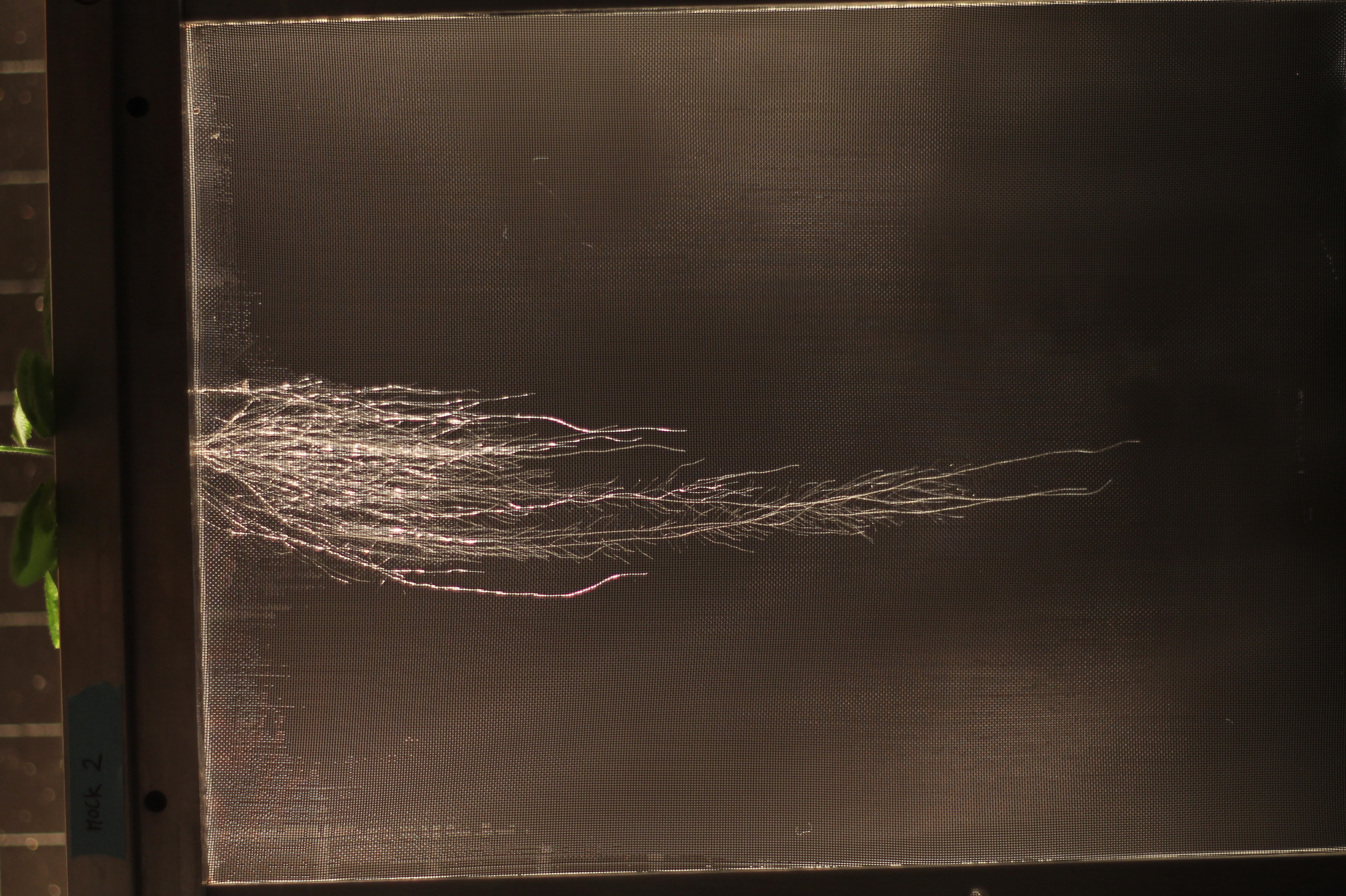

Supplement: Additional file 4: — High definition picture of RSA 13 days after the transfer (DAT). A and B,control plants. C and D,Cd-treated plants. [file 13007_2015_46_MOESM4_ESM.zip › Additional file 4 A.JPG]
